# Structural impact of arrested foveal development in children born extremely preterm without ROP at 6.5 years of age

**DOI:** 10.1038/s41433-022-02237-6

**Published:** 2022-09-16

**Authors:** Abinaya Priya Venkataraman, Zoran Popovic, Kerstin Hellgren, Johan Sjöstrand, Maria Nilsson

**Affiliations:** 1 Department of Clinical Neuroscience, Division of Eye and Vision, Unit of Optometry, Karolinska Institutet, Stockholm, Sweden; 2 Section of Ophthalmology, Department of Clinical Neuroscience, Institute of Neuroscience and Physiology, Sahlgrenska Academy, University of Gothenburg, Gothenburg, Sweden; 3 Department of Ophthalmology, Sahlgrenska University Hospital, Gothenburg, Region Västra Götaland Sweden; 4 Department of Clinical Neuroscience, Division of Eye and Vision, Karolinska Institutet, Stockholm, Sweden; 5 Astrid Lindgren Children’s Hospital, Karolinska University Hospital, Stockholm, Sweden

**Keywords:** Retinal diseases, Medical research

## Abstract

**Objectives:**

To characterize changes of foveal topography and microstructure of persisting foveal immaturity at 6.5 years of age in children born extremely preterm without retinopathy of prematurity (EPT-NoROP).

**Methods:**

Images from previous optical coherence tomography examinations of 37 EPT-NoROP and 92 control eyes were selected from a regional cohort of the EXPRESS (Extremely Preterm Infants in Sweden) study. Thickness of ganglion cell + inner plexiform layer (GCL+), outer nuclear layer (ONL), retinal thickness (RT) at the foveal centre (FC), foveal depth (FD) and RT at the foveal rim were evaluated.

**Results:**

Layer thickness of GCL+, ONL and RT was increased at FC in the EPT-NoROP group. More than two-thirds had thickness values above the control limit (control mean +2 SD) at FC (GCL + 68%, ONL 76%, and RT 68%), and 50% had reduced FD compared to controls. All parameters showed a high correlation within the EPT-NoROP group, whereas no or weaker correlation was seen in control eyes. The EPT-NoROP sub-groups, divided based on the control limit, did not differ in terms of associated factors such as gestational age, birth weight, visual acuity, and refraction.

**Conclusions:**

Extreme prematurity without impact of ROP is associated with increased GCL + , ONL, and RT thickness at FC as well as reduced FD compared to full-term controls at age 6.5. This indicates that prematurity per se may have a profound effect on foveal anatomical maturation during the first months after birth. Our results suggest RT at FC to be a simple and useful measure of foveal anatomical immaturity.

## Introduction

Preterm birth affects foveal development, and the microstructural deviations associated with low gestational age can be described using optical coherence tomography (OCT) [1–5]. These deviations include increased retinal thickness (RT) at the foveal centre (FC), decreased foveal depth (FD), incomplete migration of inner retinal layers (IRL) and abnormal formation of the outer nuclear layer (ONL) at FC [6–8]. The presence of IRL and increased ONL thickness at FC have been associated with an incomplete migration of IRL from the fovea. An increase in the combined thickness of the ganglion cell layer (GCL) and inner plexiform layer (IPL) (GCL+) has also been found [3–5].

Although these deviations are related to retinopathy of prematurity (ROP) and ROP treatment, such signs of foveal immaturity also exist in prematurely born children without ROP. Since extreme prematurity, i.e., birth before 27 gestational weeks, is highly related to ROP [3, 7, 9–11] there are limited possibilities to study the isolated impact of prematurity on foveal development in this specific group. In a previous study, including a limited number of subjects, we have shown that extremely preterm (EPT) born children have similar foveal immaturity regardless of ROP [8]. Some of the previous studies have also presented examples of cases with an underdeveloped fovea in early preterms without ROP [2, 4].

It remains to be investigated whether any OCT parameters, or relations between such parameters, can be used as markers of abnormal foveal maturation in clinical studies. For this reason, we used data from a previous study [3] and focused on aspects of persisting foveal immaturity in extremely preterm born children with no ROP (EPT-NoROP) to explore the impact of extreme prematurity per se. There is a sizeable normal variation in the foveal shape and structure [12], and this factor must be considered to quantitatively delineate preterm with abnormal or normal foveal development. OCT scans from examinations of children aged 6.5 years were analysed in order to identify OCT parameters characterizing foveal topography and microstructural deviation and to estimate the use of selected OCT parameters as markers of abnormal foveal development.

The aims of the present study are (a) to evaluate the changes and relationships of foveal OCT parameters reflecting topography and microstructure in EPT-NoROP compared to controls, (b) to estimate the proportion of EPT-NoROP eyes that are within normal limits at the foveal centre, and (c) to analyse correlations between OCT parameters at the foveal centre.

## Methods

### Study population

This study was approved by the local ethics committee and it was performed according to the terms of the Declaration of Helsinki. Written parental consent was mandatory for the participation of all children.

The OCT dataset was selected from our previously published study [3] including children born extremely preterm (EPT), i.e., before gestational age 27 weeks, in Stockholm, Sweden between 2004–2007 and age-matched controls. The cohort was drawn from the national population-based follow-up study of children born EPT, the Extremely Preterm Infants in Sweden Study (EXPRESS group) [13–15]. OCT data from examinations of 89 extremely preterm (EPT) born children and 94 age-matched controls were reviewed and left eyes of controls (*n* = 92, 39 boys and 53 girls) and both eyes of EPT-NoROP (*n* = 37 from 21 individuals, 12 girls and 9 boys) were included in this study. In the EPT-NoROP group, 6 children were born at gestational age of 25 weeks and the rest were born at gestational age of 26 weeks.

### Optical coherence tomography

Images from children examined with spectral domain Cirrus HD-OCT (Carl Zeiss Meditec, Inc, Dublin, CA) using the macular cube protocol 512 × 128 was used. One B-scan containing the foveal data was selected for further analysis. The criteria for acceptable B-scans were a signal strength ≥6, a visible foveal reflex, and/or where the maximum foveal depression was found, as well as the highest peak of the foveal photoreceptor inner segment/outer segment (IS/OS) layer is seen.

Layer segmentation was performed with a customized MATLAB program. The choroid and retinal pigment epithelium (RPE) were delineated by manually selecting points along this boundary. Images were then flattened to the posterior RPE boundary to avoid any artifacts in thickness measurements. The combined GCL + layers and the IS/OS peak were marked manually. From this measuring point, the nasal and temporal rim positions were identified automatically based on the positions of the IS/OS peak and the maximum GCL + thickness. The rim was defined as the point where the GCL + thickness reached its maximum (foveal wall maximum, FWM). Foveal depth (FD) was calculated as the difference between the mean nasal and temporal FWM thickness and retinal thickness at the foveal centre (RT@FC). From these, the following parameters were extracted: FD, GCL + thickness at FC (GCL + @FC), average GCL + thickness at the nasal and temporal quarters of the foveal rim (GCL + @Q2Q), ONL thickness, retinal thickness at FC (ONL@FC and RT@FC) and RT@FWM.

### Statistical analysis

Shapiro–Wilk was used for test of normality. Control limits were calculated based on mean values from controls and 2 standard deviations. Pearson correlation coefficient was calculated to study the relation between foveal parameters within the EPT-NoROP and controls. Independent *t*-test and Welch´s correction was used for comparison of factors associated with prematurity between EPT-NoROP within and outside limit. Statistical analysis was performed with JASP (version 0.11.1, JASP Team (2019), University of Amsterdam, Netherlands).

## Results

### Changes of foveal OCT parameters reflecting topography changes and microstructure deviations

Retinal layer thickness parameters showed a wider range and were increased in two-third of the eyes the EPT-NoROP eyes compared to the controls (Table 1). Both the combined inner retinal layers (GCL + IPL) and the outer retinal layer containing the photoreceptor cell bodies (ONL) were thicker in the EPT-NOROP group.

**Table 1:** OCT parameters in controls and EPT-NoROP.

OCT parameters	Range		Mean ± SD		Control limits (µm)	% of NoROP outside limits
	Controls (µm)	EPT-NoROP (µm)	Controls (µm)	EPT-NoROP (µm)		
GCL + @FC	0–9.1	0–42.12	3.47 ± 2.11	13.95 ± 11.66	<7.7	68%
GCL + @Q2Q	2.09–21.30	11.06–55.01	10.23 ± 4.76	27.19 ± 11.59	<19.7	73%
ONL@FC	84.85–141.63	104.49–208.66	107.78 ± 12.43	150.52 ± 26.75	<136.9	76%
RT@FC	182.48–238.75	192.72–303.95	208.36 ± 12.53	247.09 ± 4.49	<233.4	68%
FD	90.9–173.6	38.40–126.59	131.15 ± 16.73	89.46 ± 22.98	>97.7	51%

Control values are calculated based on mean values from controls and 2 standard deviations. For FD; −2SD was used. For all other parameters; +2 SD was used. *EPT-NoROP* extremely preterm born with no retinopathy of prematurity, *FD* foveal depth, *GCL+* ganglion cell layer + inner plexiform layer, *ONL* outer nuclear layer, *RT* retinal thickness, *@FC* at foveal center, *Q2Q* temporal quarter to nasal quarter distance from FC.

The proportion of GCL + ONL layers to RT@FC was 0.66 ± 0.08 and 0.53 ± 0.04 in the EPT-NoROP eyes and control eyes respectively. Individually, the proportion of ONL and GCL + layers to RT@FC was 0.6 ± 0.04 and 0.06 ± 0.04 in the EPT-NoROP group whereas the corresponding values were 0.51 ± 0.03 and 0.02 ± 0.01 for the control eyes.

The EPT-NoROP sub-groups, divided based on the control limit (within and outside limits) for RT@FC, did not show any difference in the associated factors such as gestational age, birth weight, visual acuity, and refraction (Table 2).

**Table 2:** Comparison of the associated factors, visual acuity and refractive error in EPT-No ROP groups divided based on control limits for RT@FC.

	EPT-NoROP Outside limits (*n* = 12)	EPT-NoROP Within limits (*n* = 7)	*p*-value
Gestational age (weeks)	25.83 ± 0.39	25.71 ± 0.49	0.591
Gestational age (days)	185.83 ± 3.13	184.57 ± 3.87	0.376
Birth weight (grams)	899.66 ± 170.27	875.42 ± 95.23	0.554
Visual acuity (decimal)	0.81 ± 0.17	0.83 ± 0.13	0.891
Refraction (SE, in diopters)	1.41 ± 1.04	0.84 ± 0.84	0.307

Analysis including only right eyes. *EPT-NoROP* extremely preterm born children with no retinopathy of prematurity.

### Relationship between different OCT parameters

**Fig. 1:**
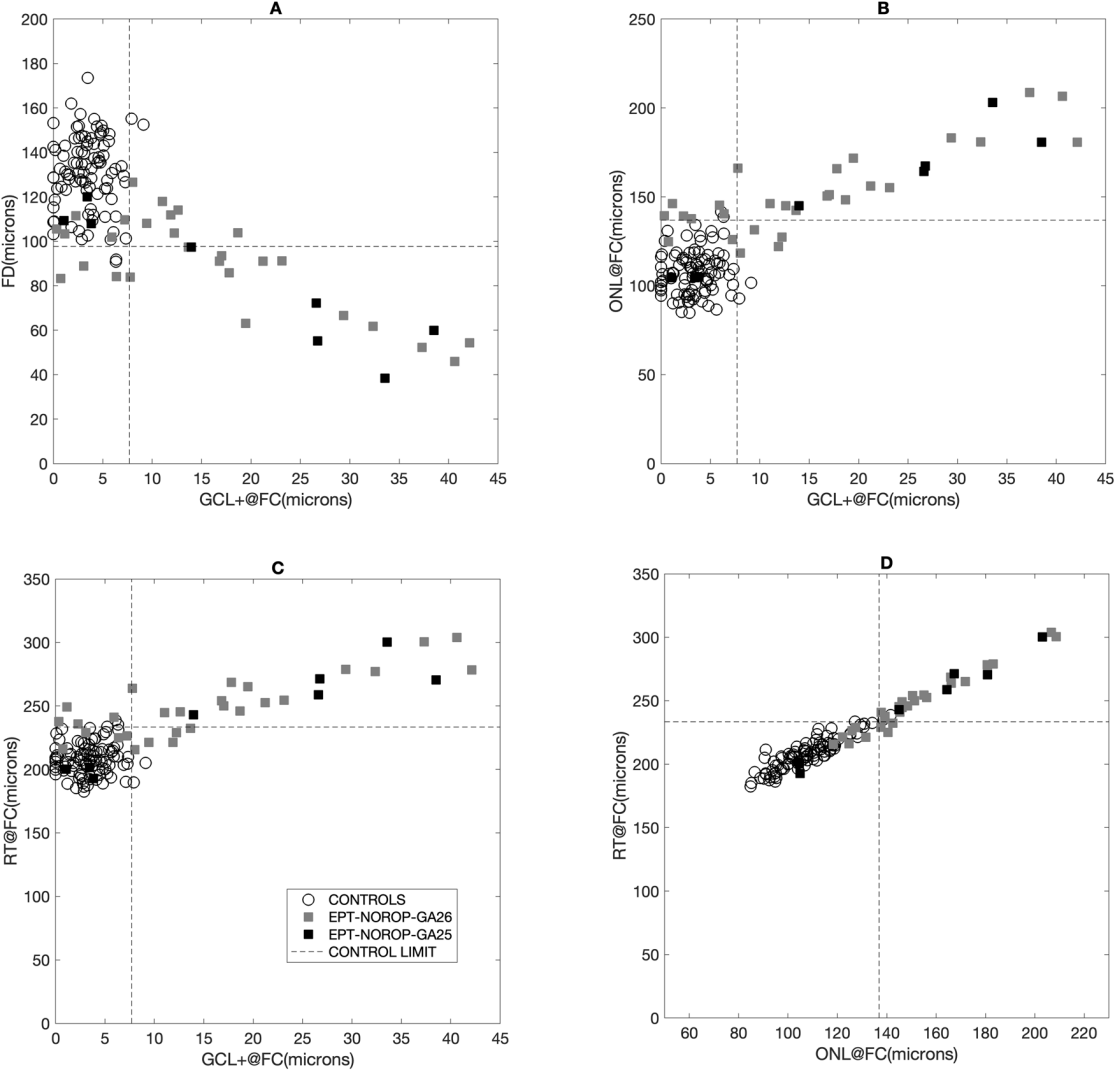
Foveal OCT parameters in Extremely preterm born children with no ROP and in control eyes. Control limits are calculated based on mean values from controls and 2 standard deviations and marked with dashed lines. EPT-NoROP: extremely preterm born children with no retinopathy of prematurity, GA Gestational age. **A** Shows the relation between foveal depth (FD) and ganglion cell + inner plexiform layer (GCL + ) at the foveal centre (FC). **B** Shows the relation between the outer nuclear layer (ONL) at the foveal centre (FC) and the ganglion cell + inner plexiform layer (GCL + ) at FC. **C** Shows the relation between the retinal thickness (RT) at the foveal centre (FC) and the ganglion cell + inner plexiform layer (GCL + ) at FC. **D** Shows the relation between the retinal thickness (RT) at the foveal centre (FC) and the outer nuclear layer (ONL) at FC.

**Fig. 2:**
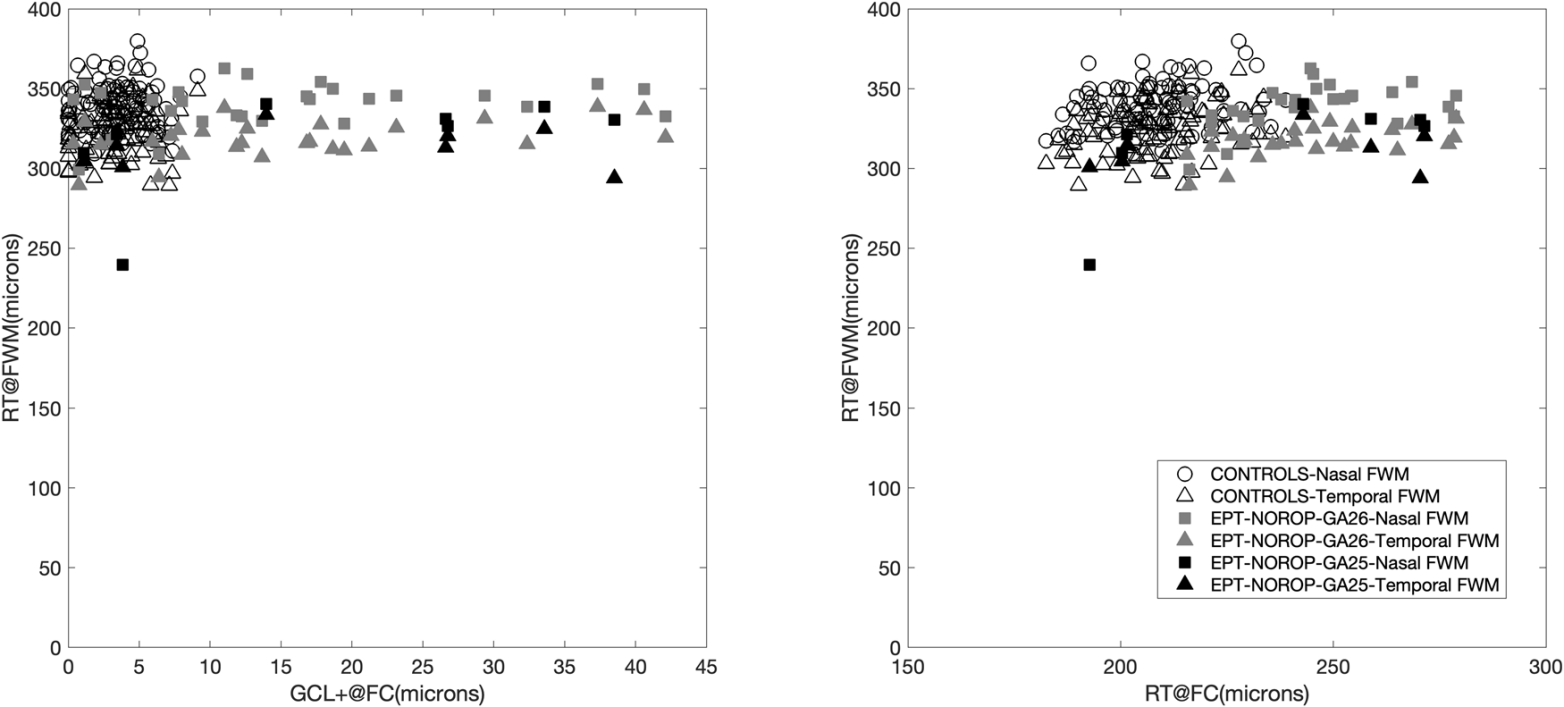
Retinal thickness at foveal rim. RT@FWM: retinal thickness at foveal wall maximum, GCL + @FC: ganglion cell layer + inner plexiform layer at foveal centre, and RT@FC: retinal thickness at foveal centre, EPT-NoROP: extremely preterm born children with no retinopathy of prematurity.

**Fig. 3:**
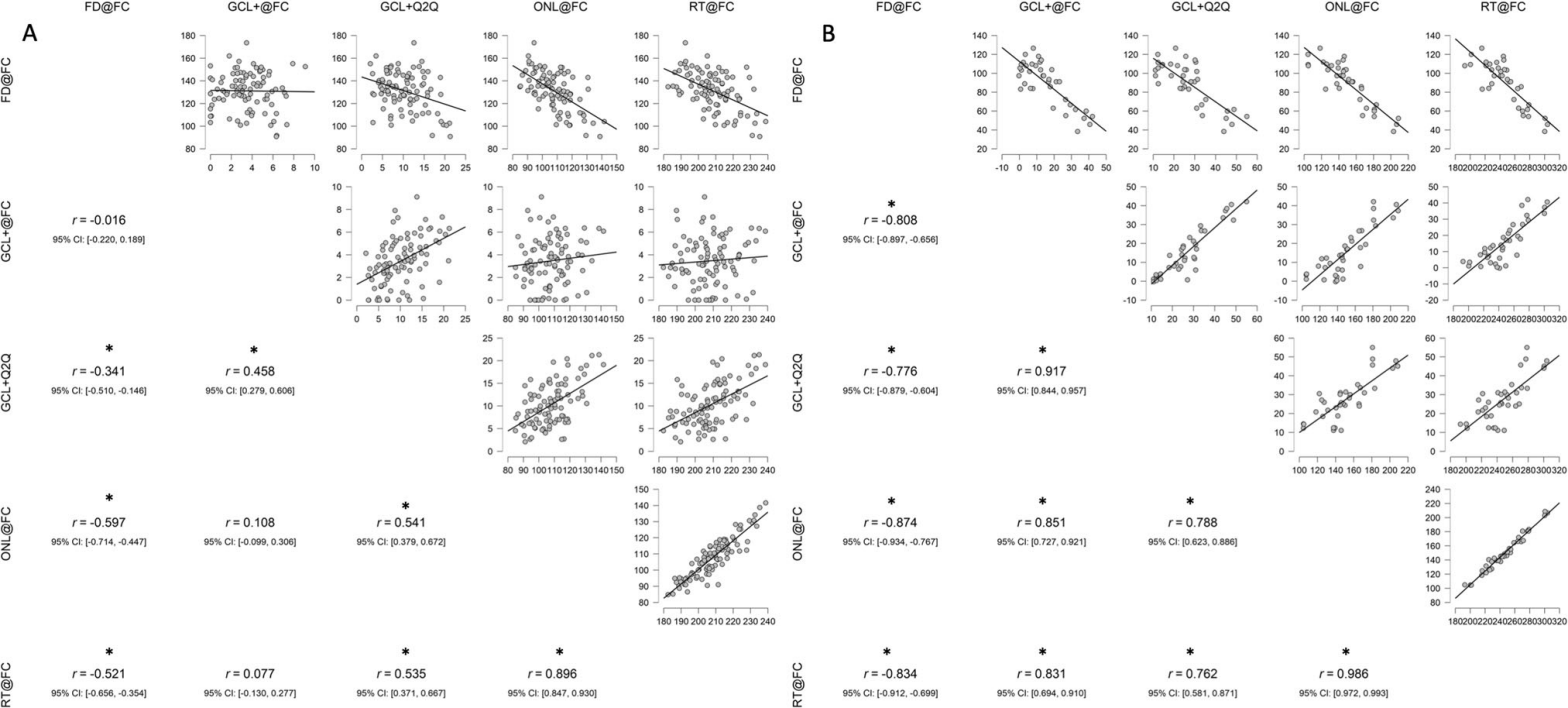
Relation between various foveal OCT parameters. Scatter plots showing the relation between various foveal OCT parameters in control eyes (**A**) and in EPT-NoROP eyes (**B**). FC Foveal centre, FD foveal depth, GCL + : ganglion cell + inner plexiform layer, Q2Q quarter to quarter distance from FC, ONL outer nuclear layer, RT retinal thickness. EPT-NoROP extremely preterm born children with no retinopathy of prematurity. Pearson’s correlation coefficient values are reported and the * indicates significant correlation (*p* < 0.001).

As the level of GCL + in EPT-NoROP eyes increases above the control limits, the foveal depth shows a corresponding decrease independent of the GA (Fig. 1A). An increase in GCL + @FC in EPT-NoROP eyes was also related to an increase in ONL layer thickness (Fig. 1B) and total retinal thickness at FC (Fig. 1C). Figure 1D shows the strong relationship between ONL@FC and RT@FC in both groups. The EPT-NoROP eyes showed larger values for both parameters. The relation between FD and GCL + @Q2Q (Fig. S1A) was similar to that of FD versus GCL + @FC (Fig. 1A). The increase in RT@FC showed a corresponding decrease in FD (Fig. S1B). Increased GCL@FC and RT@FC in the EPT-NoROP group did not show any impact on the RT@FM (Fig. 2). Within the EPT-NoROP group we found strong significant correlations between all OCT parameters, whereas that was not the case for the control group (Fig. 3). The strongest correlation was seen between ONL@FC and RT@FC for both the groups.

## Discussion

Abnormalities of the foveal structure after a preterm birth have been well described since more than a decade [1, 2, 7, 11, 16–18]. In this study we exclusively evaluated the eyes without history of ROP in a cohort of children born extremely preterm (GA < 27 weeks). Our results show that majority of these EPT-NoROP eyes had several signs of foveal immaturity as compared to full-term controls.

Typical signs of foveal immaturity found in EPT-NoROP were increased thickness of GCL + and ONL layers centrally in two thirds of the cases. The increase of IRL layers at FC seems to be the hallmark of persistent structural changes observed in preterms [1, 4, 19, 20]. The presence of IRL in the foveal center has been interpreted as a result of partial arrest of centrifugal migration which normally occurs from the 24th week of gestation [21, 22]. Half of the EPT-NoROP eyes also showed a decrease in FD. When comparing our measures with another study where EPT-NoROP eyes were analyzed, the mean values of FD and RT were nearly identical (FD; 89 compared to 85 µm, RT@FC; 247 compared to 253 µm) [4]. However, that study only included four EPT-NoROP and the EPT with history of ROP showed a larger reduction in FD and an increased RT at FC, suggesting an impact of ROP on the outcome on these specific parameters. The presence of GCL + layer at FC and its strong correlation to the other parameters characterizes and quantifies the degree of immaturity of the EPT-NOROP eyes.

The significant correlations found between all FC parameters within the EPT-NoROP group indicates that prematurity affects the different cell processes during foveal maturation in a coordinated way. A possible scenario in the preterm fovea is that the retarded centrifugal migration of inner retinal cells [22] out from FC is an early event followed by a secondary effect on the centripetal displacement of photoreceptor cell bodies. The correlations within the control group were weaker and the GCL + showed no association with the other parameters as a fully matured fovea has no IRL at FC. ONL is the main contributor to the total RT at FC and both groups showed similar and strongest correlation between ONL and RT at FC. Despite of the increased RT@FC, the RT@FWM was very similar between EPT-NoROP and controls. This indicates that the changes in foveal microstructure caused by prematurity is limited to the central fovea in NoROP-EPT and this finding was also reported in previous studies of preterms with or without ROP [4, 8, 23].

Our results indicate that both GCL + and ONL individually contributed to the increase in RT@FC. The increased thickness of the ONL in EPT-NoROP eyes is likely linked to the inhibited migration of ganglion cells [23]. The shape of the ONL after a preterm birth has been found to be higher and steeper compared to controls and in cases without history of ROP [8, 11, 23]. Our group has previously speculated that the centrally increased thickness of the ONL is related to the decreased displacement of ganglion and bipolar cells, and that the inhibited migration of ganglion cells decreases the centrifugal pull on the synaptically connected cone pedicles, bipolar cells and ganglion cells [23]. Akula et al. [11] could not associate the increased ONL with increased packing or density of the most central cones. We did not find any significant difference in the visual acuity or other associated parameters (Table 2) between the EPT-NoROP eyes that were within and outside the control limits based on RT. This is in line with what we presented in a previous study where this group of children was part of a larger cohort, including children with ROP. We then found no association between visual acuity and central foveal thickness [24].

As a single marker, GCL + @FC is a strong indicator of arrested foveal development in our study. Based on the control limits of ONL@FC, the largest proportion in the EPT-NoROP eyes were outside the limit. Interestingly, Balasubramanian and coworkers [18] found that increased GCL and ONL thickness of the central fovea was associated with worse visual acuity in young adults born extremely preterm, although most noticeable in eyes treated for ROP. There are limitations in the measurement of such thin structure as GCL@FC and to define borders of separate layers like ONL@FC. The automated segmentation algorithm used in the OCT instruments often fails to delineate individual retinal layers in eyes with altered retinal morphology [25]. Since RT@FC correlates strongly with the other parameters investigated, we suggest that RT@FC can be used as an indirect measure of arrested foveal development. Measuring RT@FC will also be robust as this is not affected by the segmentation errors as much as the individual layer measurement. As an alternative, we can also use the RT value automatically reported from the central sector of the macular thickness volumetric map. However, it might be challenging to get a reliable volumetric thickness measurements in children.

The normal variation in the foveal shape and structure [12, 26], has to be considered when quantifying the deviations of foveal development in preterms. Hence, we identified the proportion of EPT-NoROP eyes that were within and outside 2 standard deviations of the thickness values of control eyes. Though the majority of the EPT-NoROP had values outside the control limits, there was no difference in the associated factor and clinical findings between the EPT-NoROP within and outside the limits. Nevertheless, these limits that define abnormal values can be valuable for future comparisons.

In conclusion, extreme prematurity without impact of ROP is associated with increased RT, ONL and GCL + thickness at FC, with about two-third of eyes having thickness values outside the control limits. Increased GCL + thickness at the foveal center is a strong indicator of foveal immaturity. Retinal thickness at the foveal center can be used as a simple substitute measure to indicate foveal immaturity. No negative impact was seen on visual acuity due to the increased retinal layer thickness in the fovea.

## Summary

### What was known before

- Retinopathy of prematurity is associated with abnormal foveal development.
- The failure of inner retinal layers to migrate away from the fovea results in increased foveal thickness in Retinopathy of prematurity.

### What this study adds

- Foveal immaturity is also seen in eyes without history of retinopathy of prematurity after extreme preterm birth.
- The retinal thickness at the foveal centre can be used as a simple substitute measure to indicate foveal immaturity.
- Prematurity per se may have a profound effect on foveal maturation during the first months after birth.

## Supplementary information

Supplementary figure legend

Supplementary figure
