# Supplementary material for: Structural impact of arrested foveal development in children born extremely preterm without ROP at 6.5 years of age
Source: Eye (Lond). 2022 Sep 16;37(9):1810–5. doi: 10.1038/s41433-022-02237-6 (PMC10276016; doi:10.1038/s41433-022-02237-6)
Supplement: Supplementary file 1 — Supplementary figure legend [file 41433_2022_2237_MOESM1_ESM.docx]

Figure S1: The relation between A) foveal depth (FD) and ganglion cell layer + inner plexiform layer (GCL+) thickness at quarter to quarter (Q2Q) distance from foveal center (FC), and B) FD and retinal thickness (RT) at FC. EPT-NoROP: extremely preterm born children with no retinopathy of prematurity. GA: Gestational age.
