# Supplementary figures and images for: Structural impact of arrested foveal development in children born extremely preterm without ROP at 6.5 years of age
Source: Eye (Lond). 2022 Sep 16;37(9):1810–5. doi: 10.1038/s41433-022-02237-6 (PMC10276016; doi:10.1038/s41433-022-02237-6)

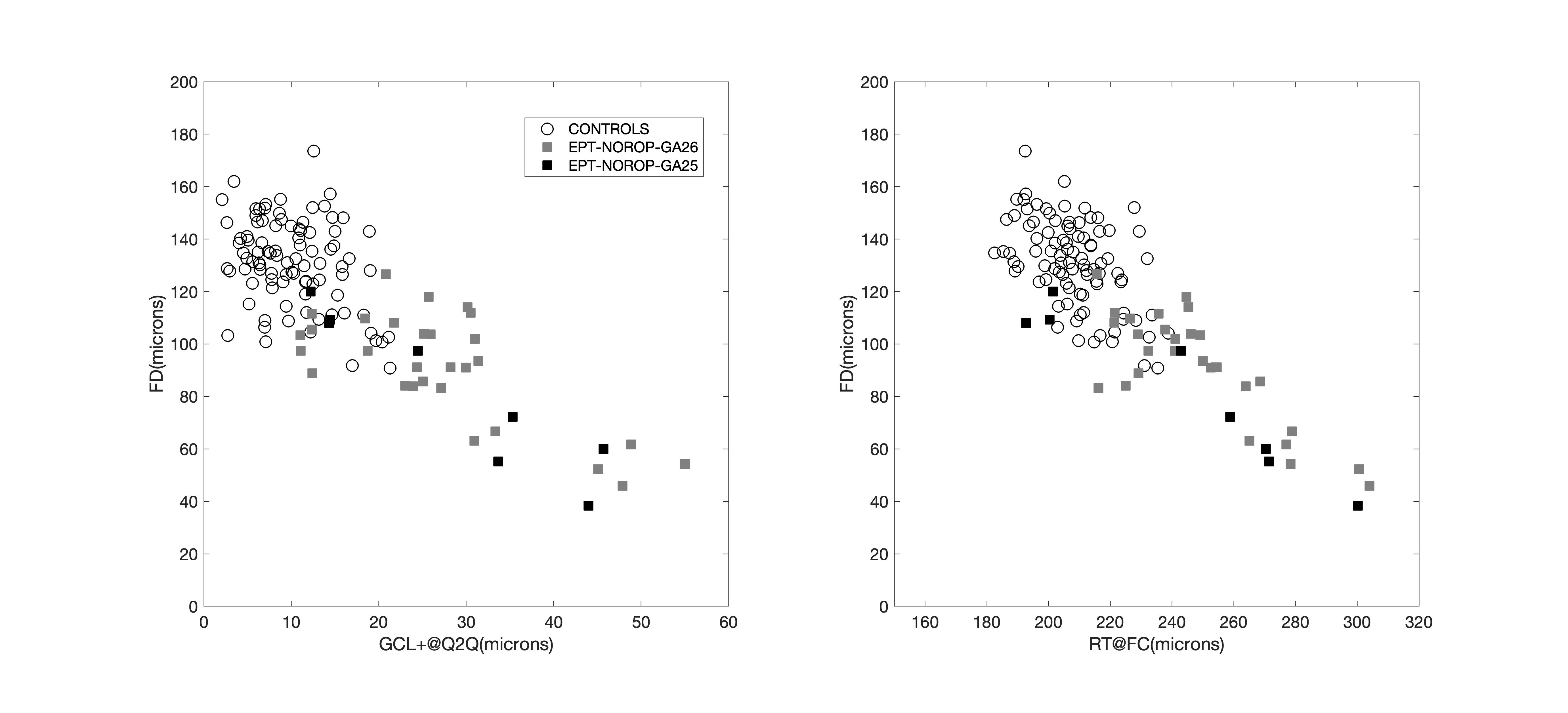

Supplement: Supplementary file 2 — Supplementary figure [file 41433_2022_2237_MOESM2_ESM.tif]
